# Rumen Biohydrogenation and Microbial Community Changes Upon Early Life Supplementation of 22:6*n*-3 Enriched Microalgae to Goats

**DOI:** 10.3389/fmicb.2018.00573

**Published:** 2018-03-27

**Authors:** Lore Dewanckele, Bruno Vlaeminck, Emma Hernandez-Sanabria, Alexis Ruiz-González, Sieglinde Debruyne, Jeyamalar Jeyanathan, Veerle Fievez

**Affiliations:** ^1^Laboratory for Animal Nutrition and Animal Product Quality, Department of Animal Sciences and Aquatic Ecology, Faculty of Bioscience Engineering, Ghent University, Ghent, Belgium; ^2^Center for Microbial Ecology and Technology, Department of Biochemical and Microbial Technology, Faculty of Bioscience Engineering, Ghent University, Ghent, Belgium; ^3^Animal Sciences Unit, Flanders Research Institute for Agriculture, Fisheries and Food, Melle, Belgium

**Keywords:** docosahexaenoic acid, early life, goat, microalgae, rumen biohydrogenation, rumen microbiome

## Abstract

Dietary supplementation of docosahexaenoic acid (DHA)-enriched products inhibits the final step of biohydrogenation in the adult rumen, resulting in the accumulation of 18:1 isomers, particularly of *trans*(*t*)-11 18:1. Occasionally, a shift toward the formation of *t*10 intermediates at the expense of *t*11 intermediates can be triggered. However, whether similar impact would occur when supplementing DHA-enriched products during pregnancy or early life remains unknown. Therefore, the current *in vivo* study aimed to investigate the effect of a nutritional intervention with DHA in the early life of goat kids on rumen biohydrogenation and microbial community. Delivery of DHA was achieved by supplementing DHA-enriched microalgae (DHA Gold) either to the maternal diet during pregnancy (prenatal) or to the diet of the young offspring (postnatal). At the age of 12 weeks, rumen fluid was sampled for analysis of long-chain fatty acids and microbial community based on bacterial 16S rRNA amplicon sequencing. Postnatal supplementation with DHA-enriched microalgae inhibited the final biohydrogenation step, as observed in adult animals. This resulted particularly in increased ruminal proportions of *t*11 18:1 rather than a shift to *t*10 intermediates, suggesting that both young and adult goats might be less prone to dietary induced shifts toward the formation of *t*10 intermediates, in comparison with cows. Although *Butyrivibrio* species have been identified as the most important biohydrogenating bacteria, this genus was more abundant when complete biohydrogenation, i.e. 18:0 formation, was inhibited. *Blautia* abundance was positively correlated with 18:0 accumulation, whereas *Lactobacillus* spp. *Dialister* spp. and *Bifidobacterium* spp. were more abundant in situations with greater *t*10 accumulation. Extensive comparisons made between current results and literature data indicate that current associations between biohydrogenation intermediates and rumen bacteria in young goats align with former observations in adult ruminants.

## Introduction

Ruminant diets commonly contain forages and concentrates. Fats are often incorporated to increase the energy density in diets for high yielding dairy cattle. Moreover, fat sources have been included in ruminant diets to increase concentrations of human-health promoting *n*-3 fatty acids (FA) and bioactive conjugated linoleic acids (CLA) in milk and meat. The majority of the dietary FA are 18-carbon unsaturated FA (linolenic acid, 18:3*n*-3; linoleic acid, 18:2*n*-6 and oleic acid, *cis*-9 18:1; Ferlay et al., 2017). However, following lipolysis, dietary unsaturated FA are converted to saturated FA in the rumen by bacteria (Buccioni et al., 2012). This process is called biohydrogenation and involves several consecutive conversions via various pathways, resulting in a plethora of FA isomers. Nevertheless, the predominant biohydrogenation pathway of 18:2*n*-6 is through its isomerization to *cis*-9, *trans*-11 CLA (*c*9, *t*11 CLA) which is further hydrogenated to *t*11 18:1 and ultimately to 18:0 (Bauman and Griinari, 2003; Harvatine et al., 2009). 18:3*n*-3 is mainly isomerized to *c*9, *t*11, *c*15 conjugated linolenic acid (CLnA) followed by a hydrogenation to *t*11, *c*15 18:2, *t*11 18:1, and ultimately to 18:0 (Shingfield and Wallace, 2014).

Regarding the potential benefits for human health, several studies investigated the effect of supplementation of poly-unsaturated FA (PUFA) in the ruminant diet (Wasowska et al., 2006; Shingfield et al., 2012; Toral et al., 2016), of which some contain docosahexaenoic acid (DHA, 22:6*n*-3). In monogastric animals and humans, feeding of such DHA-enriched supplements during pregnancy or in the early life has gained interest, as they are claimed to support vitality and growth at this young stage (Tanghe and De Smet, 2013; Brenna and Carlson, 2014). However, those supplements modify rumen biohydrogenation of 18:2*n*-6 and 18:3*n*-3 (Klein and Jenkins, 2011) as well as the rumen microbial population in adult ruminants (Boeckaert et al., 2008; Shingfield et al., 2012). Docosahexaenoic acid inhibits the final step of biohydrogenation to 18:0, which results in the accumulation of 18:1 isomers (Boeckaert et al., 2007; Zhao et al., 2016), mainly *t*11 18:1 (e.g., Shingfield et al., 2012; Zhao et al., 2016; Zhu et al., 2016). Some studies also observed an increase of *t*10 18:1 after dietary supplementation of DHA (Boeckaert et al., 2007; Shingfield et al., 2012; Zhu et al., 2016) which could indicate a shift from the main biohydrogenation pathway toward the formation of *t*10 intermediates at the expense of *t*11 intermediates in the rumen. In lactating ruminants, these intermediates (e.g., *t*10, *c*12 CLA) could inhibit milk fat synthesis in the mammary gland (Harvatine et al., 2009).

It is unknown whether similar changes occur when supplementing DHA-enriched products to ruminants at young age, during the period of rumen microbial colonization, as to our knowledge, there are no studies investigating the effect of DHA on rumen biohydrogenation using young ruminants. Therefore, the current *in vivo* study aimed to investigate the effect of a nutritional intervention with DHA in the early life of goat kids on rumen biohydrogenation and microbial community. Delivery of DHA to young animals can be either through the maternal diet (either prenatal during pregnancy or through the dam's milk) or directly through the diet of the young animal (postnatal). In the current study, we investigated the effect of prenatal and/or postnatal supplementation of DHA-enriched microalgae, which we hypothesized to induce changes in rumen microbial community and biohydrogenation, similar as in adult animals. As the microbial community in young ruminants may be less complex, it was hypothesized that the results of this experiment also could highlight a potential role of particular bacterial species in different rumen biohydrogenation steps.

## Materials and methods

### Animals, diets, and experimental design

All experimental procedures involving animals were approved by the Ethical Committee of the Faculty of Veterinary Medicine and Bioscience Engineering of Ghent University (EC2015/148). One hundred and eight Saanen dairy goats (46 multiparous and 62 primiparous goats) at similar pregnancy stage (insemination between 02/09/2015 and 25/09/2015) were selected during the last 6 weeks of pregnancy. All animals were housed in group pens and were fed, according to their maintenance requirements, grass silage (first 4 weeks) or a roughage mixture (% on DM basis: 24/60/16 of grass silage/maize silage/fodder beet; last 2 weeks) *ad libitum* supplemented with a standard concentrate (1 kg DM/day) which contained rapeseed (18.2 g/kg of fresh product). During the last 3 weeks of pregnancy, the animals were randomly divided into two experimental groups. One group (**D**^−^) received the standard concentrate during this period. The other group (**D**^+^**, prenatal treatment**) was supplemented with a DHA-enriched microalgae product, DHA Gold, replacing rapeseed (18.2 g/kg of fresh product; DHAgold^TM^, DSM Nutritional Products, Deinze, Belgium; FA composition (g/100 g fresh material): 14:0, 3.07; 16:0, 8.38; 18:0, 0.20; 18:1, 0.10; 18:2*n*-6, < 0.01; 22:5*n*-6, 5.40; 22:6*n*-3, 14.84). Both concentrates were formulated to be isoenergetic and isoproteic (Table 1). Diets were offered as two equal meals at 09h00 and 15h00. Animals had free access to fresh water.

**Table 1:** Ingredients and fatty acid composition (g/kg of fresh product) of concentrates of does.

**Ingredient**	**Concentrate**	
Soybean meal	260	
Rapeseed (D^−^) or DHA Gold (D^+^)^1^	18.2	
Maize	231.8	
Beet pulp	200	
Wheat	150	
Molasses	50	
Lignosulphonate	20	
Vitamin premix AD^2^	16	
Vitamin E^2^	1	
Feed phosphate	16	
Trace elements^3^	16	
Salt	10	
MgO 93%	7	
Calcium carbonate	4	
DVE^4^	155	
VEM^5^	920	
Fatty acid composition	D^−^	D^+^
14:0	n.d.	0.9
16:0	3.3	5.1
18:0	0.6	0.5
18:1	8.9	3.8
18:2*n*-6	9.6	8.3
22:5*n*-6	n.d.	1.1
22:6*n*-3	< 0.1	2.8

*n.d.: not detected*.

1 *D, doe; ^+^, supplemented daily with DHA Gold during the last 3 weeks of gestation (0.28 g per kg BW); ^−^, no DHA Gold supplementation*.

2 *VIT A, D and E (UI/g): A (312), D (62.5), and E (6,000)*.

3 *Premix of trace elements and minerals (mg/g): Fe (281), Cu (62.5), Mn (116), Co (1.4), Zn (175), I (4.8), Ca (244.3), P (157), K (248), Mg (73.8), and Na (8.1)*.

4 *DVE, true protein digested in the small intestine (Tamminga et al., 1994)*.

5 *VEM, feed unit milk (1,000 VEM = 6.9 MJ; Van Es, 1978)*.

**Figure 1:**
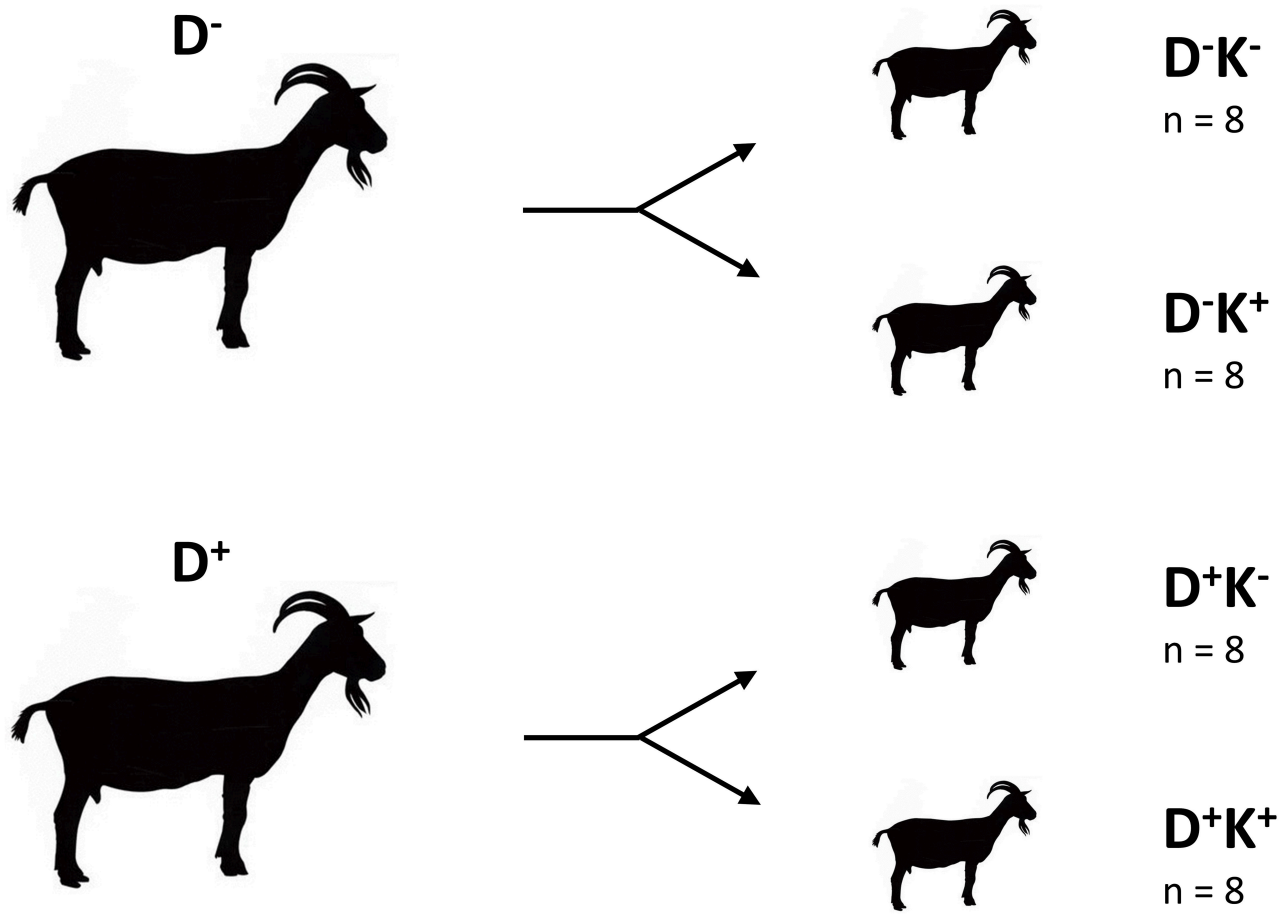
Experimental groups. D, doe; K, kid; ^+^, supplemented with DHA Gold (0.28 g per kg BW); ^−^, no DHA Gold supplementation.

After birth, only male twin goat kids were further used in the experiment. From each group of does (D^−^ and D^+^), eight male twins were selected. The twins were immediately separated from their mother and each randomly allocated to one of two experimental groups. One group (**K**^+^**, postnatal treatment**) was supplemented daily with 0.28 g DHA Gold per kg of body weight (BW) whereas the other group (**K**^−^) was not. This resulted in four experimental groups of eight kids per group (**D**^−^**K**^−^, **D**^−^**K**^+^, **D**^+^**K**^−^ and **D**^+^**K**^+^) as illustrated in Figure 1. Kids were housed in pairs of the same experimental condition in pens equipped with rubber mats and bedded with straw (1.8 × 2.2 m). The pens were constructed to avoid physical contact between the neighboring kids during the first weeks of life. Treatment (K^+^) started immediately after birth until the age of 85 ± 2 days (ca. 12 weeks old). A DHA Gold emulsion in water (0.333 g/mL) was prepared fresh and was administered orally before the morning and afternoon feeding with a 10-mL syringe. All kids received colostrum during the first 2 days after birth, which was replaced by goat milk powder from day 3 until weaning at 9 weeks. All kids received hay *ad libitum* and a standard concentrate (maximum 0.5 kg/day per pen) from week 3 onwards. From week 6 until weaning, milk powder gradually decreased and the amount of concentrate (maximum 1 kg/day per pen) increased. Diets were divided into two meals at 8h30 and 16h30. All kids had constant access to fresh water.

### Rumen sample collection

At the age of 85 ± 2 days (ca. 12 weeks old), rumen fluid was collected by stomach tube before the morning feeding after an overnight period without access to concentrate and hay. Briefly, a flexible plastic tube (15 mm i.d.) with 3 holes (± 5 mm i.d.) in the probe head was warmed up using hot water and was inserted in the rumen via the esophagus. Rumen samples were obtained using an electric vacuum pump. This method has been validated as a feasible alternative to surgical rumen cannulation in goats to examine dietary effects on the rumen FA profile and the rumen bacterial community (Ramos-Morales et al., 2014). Treated kids (K^+^) received their last supplement of DHA Gold 2 h before the sampling. An aliquot of 50 mL rumen fluid was filtered through a sieve with a pore size of 1 mm. Subsamples for analysis of long-chain FA (LCFA; 2 mL homogenized rumen fluid) were collected in glass tubes, stored at −20°C and freeze-dried prior to LCFA analysis. Subsamples for bacterial community analysis (3 mL homogenized rumen fluid) were collected in cryovials and stored at −80°C until gDNA extraction. For practical reasons, rumen sample collection was performed on two different days. On both sampling days, animals from each experimental condition were included.

### Long-chain fatty acid composition

Fatty acids were methylated as described by Vlaeminck et al. (2014). Briefly, toluene (2 mL) containing the internal standard (21:0; Sigma Aldrich, Diegem, Belgium) and methanolic NaOH (2 mL) were added and the mixture was incubated at 70°C for 60 min. This was followed by 30 min at 50°C after addition of methanolic HCl (3 mL), prepared by dissolving acetyl chloride in methanol (5/1, v/v). Fatty acid methyl esters (FAME) were extracted with hexane. Analysis of FAME was carried out using a gas chromatograph (HP7890A; Agilent Technologies, Diegem, Belgium) equipped with a SP-2560 capillary column (75 m × 0.18 mm i.d. × 0.14 μm thickness; Supelco Analytical, Bellefonte, PA, USA) and a flame ionization detector. The temperature program was as follows: initially 70°C for 2 min, increasing by 15°C/min to 150°C, followed by a second increase at 1°C/min up to 165°C and holding for 12 min, followed by a third increase at 2°C/min to 170°C, held at 170°C for 5 min, increased at 5°C/min to 215°C and held at 215°C for 20 min. Inlet and detector temperatures were 250°C and 255°C, respectively. The split ratio was 50:1. Hydrogen was used as the carrier gas at a flow rate of 1 mL/min. Identities of peaks were determined using mixtures of methyl ester standards (22:5*n*-6 and GLC463, Nu-Chek-Prep, Elysian, MN, USA; *c*9, *t*11 CLA and *t*10, *c*12 CLA, Larodan 279, Fine Chemicals AB, Malmö, Sweden). Quantification of FA was based on the area of the internal standard and on the conversion of peak areas to the weight of FA by a theoretical response factor for each FA (Ackman and Sipos, 1964; Wolff et al., 1995).

#### Statistical analysis

Data were analyzed using the MIXED procedure of SAS (version Enterprise Guide 7.1; SAS Institute Inc., Cary, NC, USA) by the following model: *Y*_*ijkl*_ = μ + *D*_*i*_ + *K*_*j*_ + *T*_*k*_ + *DO*_*l*_ + *D*_*i*_ × *K*_*j*_ + ε_*ijkl*_, with *D*_*i*_ the fixed effect of prenatal treatment (*i* = D^−^ or D^+^), *K*_*j*_ the fixed effect of postnatal treatment (*j* = K^−^ or K^+^), *T*_*k*_ the random effect of sampling day (*k* = day 1 or 2), *DO*_*l*_ the random effect of doe (*l* = doe 1 until 16), *D*_*i*_ × *K*_*j*_ the interaction between prenatal and postnatal treatment and ε_*ijkl*_ the residual error term. Least square means are reported with treatment effects declared significant at *P* < 0.05. Tukey-Kramer multiple comparison test was used to evaluate significant differences.

### Bacterial community analysis

#### DNA extraction

gDNA extraction was performed using the repeated bead beating plus column purification (RBB+C) method as described by Yu and Morrison (2004). The yield and quality of extracted DNA were determined using a NanoDrop spectrophotometer (VWR International BVBA, Leuven, Belgium).

#### Illumina library generation and data mining

Bacterial 16S rRNA amplicon sequencing (V3-V4 region) using Illumina MiSeq technology (2 × 300 bp) was performed by Macrogen Sequencing Service (Macrogen Korea, Seoul, Rep. of Korea). Preparation of the amplicon barcoded library (primers: 344F and 806R; Klindworth et al., 2013) was based on the 16S metagenomic sequencing library preparation protocol provided by the manufacturer (Illumina, https://support.illumina.com).

The amplicon sequencing dataset was demultiplexed and barcodes were clipped off by the sequence provider. Forward and reverse reads were merged using the fastq-join method (Aronesty, 2011) after which primer removal and quality filtering was performed using the open-source software package QIIME (v1.9.1; Caporaso et al., 2010). This resulted in 43 459 ± 5812 reads per sample. Rarefaction analyses were performed using the QIIME software package (Caporaso et al., 2010) indicating that the sequencing depth was sufficient to analyze the bacterial communities in all samples (data not shown). The subsequent analysis, picking Operational Taxonomic Units (OTU), assigning taxonomy, inferring phylogeny and creating OTU tables, were also performed by QIIME software (Caporaso et al., 2010). The sequences were clustered into OTU using the open-reference OTU picking workflow with a 97 % similarity threshold using UCLUST (Edgar, 2010) and chimeras were removed using UCHIME (Edgar, 2010). Representative sequences from each OTU were aligned using PyNAST (Caporaso et al., 2010) and a taxonomy identity was assigned to each representative sequence using the method UCLUST (Edgar, 2010) and the GreenGenes database for reference (v13_8; DeSantis et al., 2006). OTU with <0.005% of the total number of sequences were removed. To ensure the comparability of the species diversity between the samples, normalized/rarefied OTU sets were used for further analysis.

Alpha diversity indices (Chao1 index, Observed OTU, PD whole tree) were calculated and significant differences between experimental groups were determined by the non-parametric Kruskal-Wallis test in QIIME (Caporaso et al., 2010). Beta diversity indices between samples were determined in QIIME (Caporaso et al., 2010) based on Bray-Curtis dissimilarity (Bray and Curtis, 1957) and Unweighted UniFrac metric (Lozupone and Knight, 2005). The non-parametric permutational MANOVA-based statistical test ANOSIM was used in QIIME (Caporaso et al., 2010) to determine differences in microbial community between experimental groups. Differences in relative abundance of the different taxa (at genus level) between treatments were determined using the MIXED procedure of SAS (version Enterprise Guide 7.1; SAS Institute Inc., Cary, NC, USA) by the following model: *Y*_*ijkl*_ = μ + *D*_*i*_ + *K*_*j*_ + *T*_*k*_ + *DO*_*l*_ + *D*_*i*_ × *K*_*j*_ + ε_*ijkl*_, with *D*_*i*_ the fixed effect of prenatal treatment (*i* = D^−^ or D^+^), *K*_*j*_ the fixed effect of postnatal treatment (*j* = K^−^ or K^+^), *T*_*k*_ the random effect of sampling day (*k* = day 1 or 2), *DO*_*l*_ the random effect of doe (*l* = doe 1 until 16), *D*_*i*_ × *K*_*j*_ the interaction between prenatal and postnatal treatment and ε_*ijkl*_ the residual error term. Least square means are reported with treatment effects declared significant at *P* < 0.05 and with a trend toward significance at 0.05 ≤ *P* < 0.10. Significant differences were evaluated with the Tukey-Kramer multiple comparison test. In addition, Spearman Rank correlation was used to check the correlation between different FA and different taxa (at genus level) using QIIME (Caporaso et al., 2010).

Sequence data have been deposited in the National Center for Biotechnology Information (NCBI) database under accession number PRJNA414378.

#### Multivariate statistical analysis

A bipartite network was inferred using a similarity matrix obtained from a regularized canonical correlation analysis (rCCA), using the package mixOmics (v6.2.0; Lê Cao et al., 2011) in R (v3.4.1; Kurtz et al., 2015). In this analysis, the correlation values between the relative abundances of bacterial taxa (at genus level) and each LCFA were computed to calculate a similarity matrix. Then, these values were projected onto the space spanned by the first components retained in the analysis. Three relevant components were obtained setting a threshold to R = 0.40. Relevance networks are a robust approach to highlight functional relationships, because they simultaneously represent positive and negative correlations, which are missed by methods using Euclidian distances. Another advantage of the rCCA is its ability to represent correlations across disparate biological measures, such as the bacterial relative abundances and metabolic information (De Weirdt et al., 2017).

## Results

### Rumen fatty acid composition

Postnatal treatment with 0.28 g DHA Gold per kg BW increased the rumen proportions of 14:0 (*P* = 0.003), 16:0 (*P* < 0.001), 22:5*n*-6 (*P* < 0.001) and 22:6*n*-3 (*P* < 0.001, Table 2). This may be associated with the high amounts of these FA in the algal cell biomass. In contrast, prenatal treatment did not affect the rumen proportion of any of these FA (*P* > 0.05, Table 2).

**Table 2:** Effect of prenatal and/or postnatal treatment of goat kids with DHA Gold on proportions of long-chain fatty acids (g/100 g fatty acids) in rumen fluid.

**Fatty acid^3^**	**Experimental group^1^**				**SEM^2^**	***P*****-value**		
	**D^−^K^−^ (*n* = 8)**	**D^−^K^+^ (*n* = 8)**	**D^+^K^−^ (*n* = 8)**	**D^+^K^+^ (*n* = 8)**		**Prenatal treatment (D)**	**Postnatal treatment (K)**	**D × K**
14:0	1.58	3.33	2.59	3.35	0.478	0.203	0.003	0.178
16:0	15.31	17.59	16.01	17.38	0.667	0.570	<0.001	0.247
18:0	43.46	32.05	40.43	32.32	2.710	0.633	0.001	0.503
*t*6 18:1 + *t*7 18:1 + *t*8 18:1^*^	0.21	0.51	0.16	0.38	0.089	0.132	0.002	0.474
*t*9 18:1	0.05	0.31	0.06	0.23	0.046	0.489	<0.001	0.356
*t*10 18:1^*^	0.70	1.09	0.56	1.61	0.597	0.403	0.320	0.731
*t*11 18:1^*^	0.64	1.62	0.81	1.37	0.323	0.921	0.002	0.232
*t*12 18:1	0.15	0.54	0.10	0.32	0.078	0.106	0.002	0.272
*c*9 18:1 + *t*13 18:1 + *t*14 18:1	2.39	2.85	2.37	2.43	0.335	0.476	0.372	0.493
*c*11 18:1 + *t*15 18:1	1.23	2.03	1.58	1.59	0.246	0.850	0.123	0.131
*c*12 18:1	0.41	0.29	0.39	0.27	0.054	0.706	0.037	0.968
*c*13 18:1	0.10	0.32	0.27	0.38	0.207	0.430	0.015	0.334
*c*14 18:1 + *t*16 18:1	0.33	0.49	0.13	0.25	0.181	0.174	0.059	0.801
Sum 18:1^*^	6.31	10.14	6.35	8.74	1.271	0.380	0.018	0.372
*c*9, *t*11 CLA	0.07	0.19	0.12	0.17	0.058	0.873	0.164	0.574
*t*10, *c*12 CLA	0.01	0.01	<0.01	<0.01	0.010	0.179	0.969	0.969
18:2*n*-6	2.67	1.97	2.57	1.71	0.347	0.615	0.028	0.812
*c*9, *t*11 CLA + *t*11 18:1^*^	0.72	1.81	0.92	1.53	0.334	0.980	0.003	0.227
*t*10, *c*12 CLA + *t*10 18:1^*^	0.71	1.11	0.56	1.61	0.597	0.363	0.312	0.719
18:3*n*-3	0.59	0.43	0.70	0.56	0.075	0.157	0.038	0.884
*c*9, *t*11, *c*15 CLnA	0.09	0.15	0.10	0.06	0.034	0.350	0.673	0.058
22:5*n*-6	<0.01	1.43	0.12	1.75	0.293	0.140	<0.001	0.654
22:6*n*-3	<0.01	3.81	0.34	4.63	0.817	0.137	<0.001	0.716

1 *D, doe; K, kid; ^+^, supplemented with DHA Gold (0.28 g per kg BW); ^−^, no DHA Gold supplementation*.

2 *SEM, standard error of the mean*.

3 *t, trans; c, cis; CLA, conjugated linoleic acid; CLnA, conjugated linolenic acid*.

* *Reported P-values are the P-values from the logarithm*.

Prenatal treatment with DHA Gold did not affect the rumen proportions of 18:2*n*-6, 18:3*n*-3 and their biohydrogenation intermediates (*P* > 0.05, Table 2), whereas postnatal treatment decreased the proportions of 18:2*n*-6 (*P* = 0.028) and 18:3*n*-3 (*P* = 0.038) in the rumen. However, when expressed as μg per mL of rumen fluid (Supplementary Table 1), no significant effect of postnatal treatment on 18:2*n*-6 or 18:3*n*-3 was observed (*P* > 0.05). The rumen proportions of *t*6 18:1 + *t*7 18:1 + *t*8 18:1 (*P* = 0.002), *t*9 18:1 (*P* < 0.001), *t*11 18:1 (*P* = 0.002), *t*12 18:1 (*P* = 0.002), and *c*13 18:1 (*P* = 0.015) increased upon postnatal DHA Gold supplementation (Table 2). This resulted in a higher proportion of total 18:1 FA (*P* = 0.018), which was accompanied by a decrease in 18:0 (*P* = 0.001). In contrast to the other 18:1 FA, *c*12 18:1 decreased (*P* = 0.037) after postnatal treatment with DHA Gold. No difference between treatments was observed for *c*9, *t*11 CLA (*P* = 0.164), *t*10, *c*12 CLA (*P* = 0.969) and *c*9, *t*11, *c*15 CLnA (*P* = 0.673). In none of the treatments, *t*10, *c*12, *c*15 CLnA was detected in the rumen (data not shown).

Neither prenatal nor postnatal treatment with DHA Gold induced a shift toward the formation of *t*10 intermediates at the expense of *t*11 intermediates. Nevertheless, both *t*10 18:1 as well as *t*11 18:1 increased upon postnatal DHA Gold supplementation. However, the increase in *t*10 intermediates was not significant due to large inter-animal variation ($\bar{x}$ ± SD = 1.00 ± 1.66 g/100 g FA; SEM = 0.597 g/100 g FA).

### Rumen microbiome

**Figure 2:**
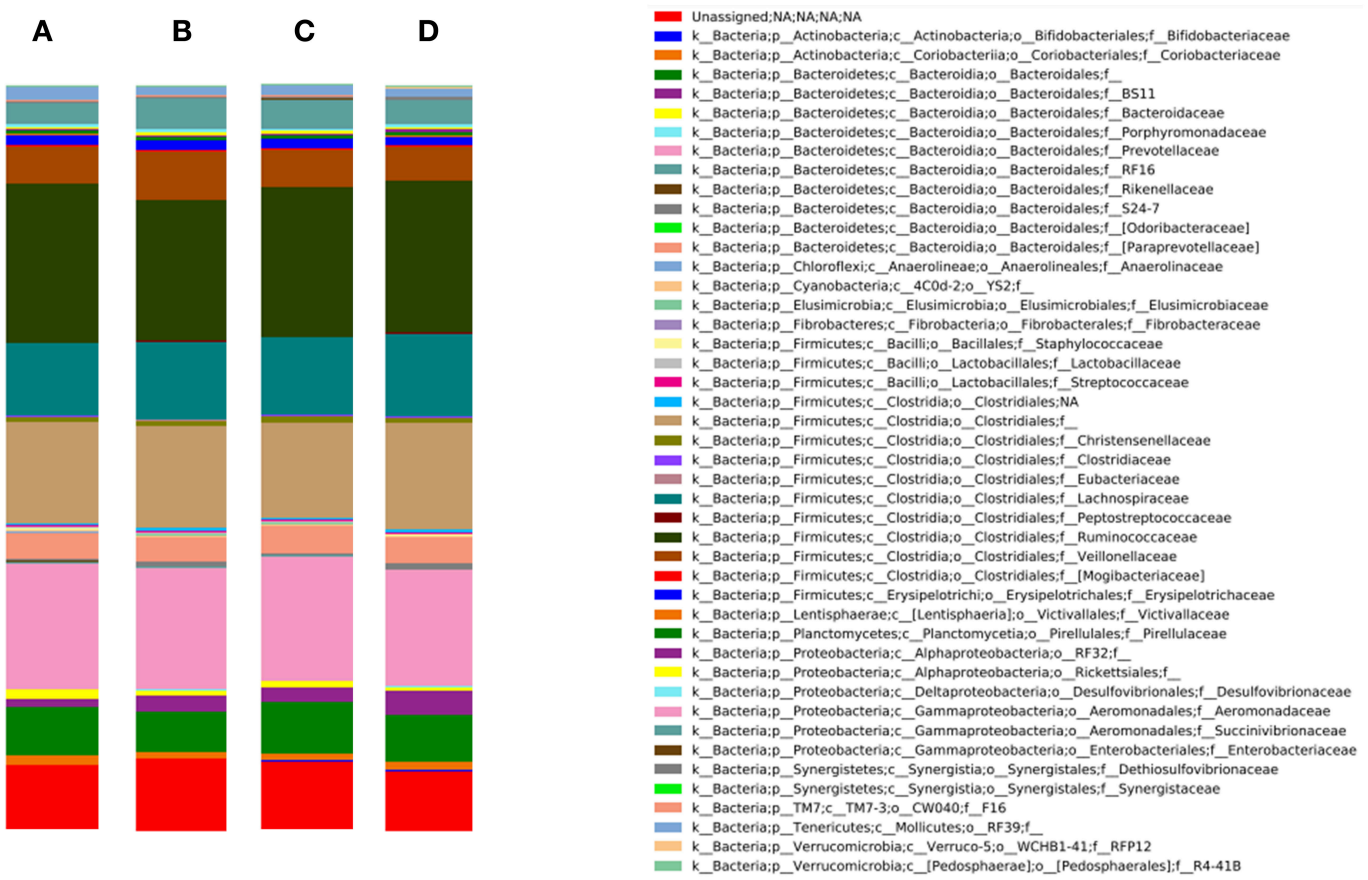
Bacterial community composition on family level in the rumen of the different experimental groups: **(A)** D^−^K^−^, **(B)** D^−^K^+^, **(C)** D^+^K^−^, and **(D)** D^+^K^+^ (*n* = 8). D, doe; K, kid; ^+^, supplemented with DHA Gold (0.28 g per kg BW); ^−^, no DHA Gold supplementation.

Figure 2 represents the bacterial community composition in the rumen of goats from the different experimental groups. The 3 most abundant phyla were Firmicutes (52.8% of relative abundance), Bacteroidetes (29.9% of relative abundance) and Proteobacteria (4.6% of relative abundance). Within the Firmicutes, the families Veillonellaceae, Ruminococcaceae (predominant genus: *Ruminococcus*) and Lachnospiraceae and undefined families within the order of the Clostridiales dominated the rumen. The predominant families of the Bacteroidetes were Prevotellaceae (predominant genus: *Prevotella*) and Paraprevotellaceae, whereas the Proteobacteria mainly consisted of undefined genera within the family Succinivibrionaceae.

Neither prenatal nor postnatal treatment influenced bacterial species richness as expressed by different alpha diversity indices (*P* > 0.10; Supplementary Figure 1). Nevertheless, the statistical test ANOSIM revealed differences in microbial community between experimental groups (*P*_Bray−Curtis_ = 0.011; *P*_UnweightedUniFrac_ = 0.016). Taxa with significant differences or with a trend toward significance upon prenatal and/or postnatal treatment with DHA Gold are shown in Table 3. Prenatal treatment increased or tended to increase the relative abundance of the families RF16 (*P* = 0.020) and BS11 (*P* = 0.075) within the order of the Bacteroidales and the genera *Clostridium* (*P* = 0.009) and *Butyrivibrio* (*P* = 0.079) within the order of the Clostridiales. Postnatal treatment increased the relative abundance of the families BS11 (*P* = 0.038) and S24-7 (*P* = 0.006) within the order of the Bacteroidales and of *Butyrivibrio* species (*P* = 0.007) and the family Veillonellaceae (*P* = 0.011; order Clostridiales) whereas it decreased the relative abundance of BF311 (*P* = 0.033; order Bacteroidales), *Blautia* species (*P* = 0.028; order Clostridiales), RF39 (*P* = 0.016) and YS2 (*P* = 0.025). Besides this, interaction effects between prenatal and postnatal treatment were observed for some taxa. Postnatal treatment altered (*P* < 0.05) the relative abundance of some taxa, but only when does were not treated with DHA Gold (YRC22, Ruminococcaceae, *Acidaminococcus, Selenomonas, Succiniclasticum*, L7A_E11).

**Table 3:** Average relative abundance (%) of different bacterial taxa in the rumen of goat kids supplemented pre- and/or postnatally with DHA Gold.

**Taxon: order/family/genus^3^**	**Experimental group^1^**				**SEM^2^**	***P*****-value**		
	**D^−^K^−^ (*n* = 8)**	**D^−^K^+^ (*n* = 8)**	**D^+^K^−^ (*n* = 8)**	**D^+^K^+^ (*n* = 8)**		**Prenatal treatment (D)**	**Postnatal treatment (K)**	**D × K**
Aeromonadales/Succinivibrionaceae	2.28^B^	3.59^A^	3.44^AB^	2.93^AB^	0.506	0.624	0.449	0.093
Anaerolineales/Anaerolinaceae/SHD-231	<0.01	0.06	0.03	0.10	0.032	0.334	0.060	0.910
Bacteroidales/Bacteroidaceae/BF311	0.48	0.28	0.34	0.21	0.069	0.147	0.033	0.608
Bacteroidales/BS11	0.87	2.13	1.94	3.01	0.508	0.075	0.038	0.854
Bacteroidales/Paraprevotellaceae/YRC22	0.37^b^	0.72^a^	0.55^ab^	0.45^ab^	0.147	0.654	0.189	0.027
Bacteroidales/RF16	0.08	0.02	0.18	0.16	0.060	0.020	0.233	0.632
Bacteroidales/S24-7	0.28	0.49	0.39	0.72	0.111	0.111	0.006	0.490
Clostridiales	0.40	0.49	0.30	0.41	0.054	0.138	0.091	0.876
Clostridiales/Clostridiaceae/*Clostridium*	0.03	<0.01	0.18	0.21	0.152	0.009	0.836	0.538
Clostridiales/Lachnospiraceae/*Blautia*	0.45	0.21	0.37	0.27	0.071	0.862	0.028	0.368
Clostridiales/Lachnospiraceae/*Butyrivibrio*	1.88	2.67	2.38	3.17	0.258	0.079	0.007	0.996
Clostridiales/Ruminococcaceae	15.70^a^	13.85^b^	13.71^b^	14.12^b^	0.664	0.075	0.089	0.013
Clostridiales/Veillonellaceae	2.15	3.26	2.02	2.21	0.323	0.168	0.011	0.055
Clostridiales/Veillonellaceae/*Acidaminococcus*	0.31^a^	0.17^b^	0.13^b^	0.18^ab^	0.059	0.210	0.082	0.004
Clostridiales/Veillonellaceae/*Selenomonas*	0.02^b^	0.12^a^	0.02^b^	0.05^b^	0.019	0.135	<!0.001	0.048
Clostridiales/Veillonellaceae/*Succiniclasticum*	0.79^b^	1.25^a^	1.17^a^	1.02^ab^	0.098	0.462	0.127	0.007
Desulfovibrionales/Desulfovibrionaceae	0.04	0.08	0.03	0.07	0.022	0.795	0.079	0.862
Enterobacteriales/Enterobacteriaceae/NA	0.01^b^	0.02^ab^	0.05^a^	<0.01^b^	0.011	0.435	0.100	0.023
Erysipelotrichales/Erysipelotrichaceae/L7A_E11	0.04^b^	0.12^a^	0.10^a^	0.10^a^	0.037	0.504	0.041	0.042
Pirellulales/Pirellulaceae	0.43^A^	0.32^B^	0.32^AB^	0.40^AB^	0.047	0.717	0.540	0.067
RF39	1.73	1.08	1.33	1.15	0.186	0.388	0.016	0.141
Synergistales/Synergistaceae/NA	0.09	0.04	0.02	<0.01	0.028	0.191	0.031	0.296
YS2	0.35	0.21	0.39	0.17	0.073	0.984	0.025	0.567

*Only taxa with significant differences (P < 0.05) or with a trend toward significance (0.05 ≤ P < 0.10) are shown*.

1 *D, doe; K, kid; ^+^, supplemented with DHA Gold (0.28 g per kg BW); ^−^, no DHA Gold supplementation*.

2 *SEM, standard error of the mean*.

3 *NA, not assigned*.

a, b *Means annotated with a different letter differ (P < 0.05) between experimental groups*.

A, B *Means annotated with a different capital letter tend to differ (0.05 ≤ P < 0.10) between experimental groups*.

### Correlation between important 18-carbon fatty acids and microbial population in the rumen

**Figure 3:**
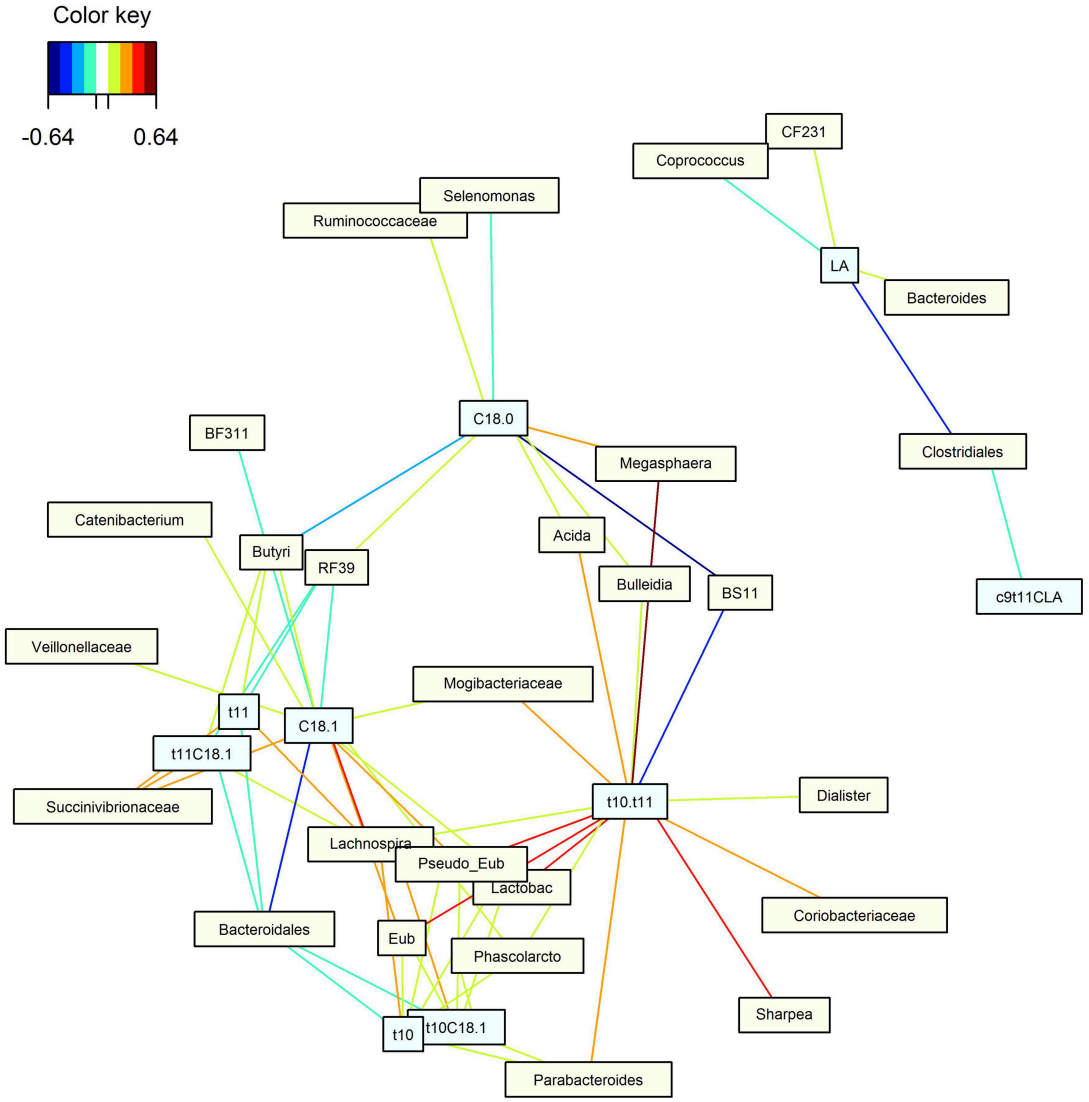
Relationships among clusters of bacterial genera and 18-carbon fatty acids irrespective of experimental group. This bipartite network was based on the regularized canonical correlations between relative bacterial abundances and relative concentrations of 18-carbon fatty acids. Interactions have been filtered for an absolute correlation above 0.4 and are colored following the key shown. This representation uncovers potentially functional populations. LA, 18:2*n*-6; c9t11CLA, *cis*-9, *trans*-11 conjugated linoleic acid; t11C18:1, *trans*-11 18:1; t11, total *trans*-11 fatty acids; C18.1, total 18:1 fatty acids; t10, total *trans*-10 fatty acids; t10C18:1, *trans*-10 18:1; t10.t11, ratio of *trans*-10 to *trans*-11 intermediates; C18.0, 18:0. Acida, *Acidaminococcus*, Butyri, *Butyrivibrio*, Eub, *Eubacterium*, Lactobac, *Lactobacillus*, Phascolarcto, *Phascolarctobacterium*, Pseudo_Eub, *Pseudoramibacter eubacterium*. No correlations were found with 18:3*n*-3 and *cis*-9, *trans*-11, *cis*-15 conjugated linolenic acid.

Two different approaches were used to investigate correlations between 18:2*n*-6, 18:3*n*-3 or their biohydrogenation intermediates and bacterial taxa. Spearman Rank correlations were calculated in QIIME and are presented in Table 4. Secondly, a bipartite network was inferred using a similarity matrix obtained from a regularized canonical correlation analysis (rCCA; Figure 3). Although most of the significant correlations were rather weak (|R| < 0.50), some stronger correlations were found. Undefined genera of the order Clostridiales were found to be negatively correlated with 18:2*n*-6 (Figure 3) whereas the family S24-7 within the order of the Bacteroidales was found to be negatively correlated with 18:3*n*-3 (Table 4). *Acidaminococcus*, RF16, Ruminococcaceae and BF311 were correlated with *t*11 intermediates based on Spearman Rank correlation (Table 4), however no strong correlations with these taxa were observed in Figure 3. *Bifidobacterium* (Table 4), *Dialister* (Table 4), *Megasphaera* (Table 4, Figure 3), Coriobacteriaceae (Table 4, Figure 3), *Lactobacillus* (Table 4, Figure 3), *Sharpea* (Figure 3), *Pseudoramibacter eubacterium* (Figure 3) and *Eubacterium* (Figure 3) were positively correlated with *t*10 intermediates or the ratio of *t*10 to *t*11 intermediates whereas Pirellulaceae (Table 4), Rickettsiales (Table 4) and BS11 (Figure 3) correlated negatively with these intermediates. The family Succinivibrionaceae within the order of the Aeromonadales (Table 4, Figure 3) and the genus *Lachnospira* (Figure 3) correlated positively with 18:1 FA whereas unknown genera within the order of the Bacteroidales correlated negatively with 18:1 FA (Figure 3). Besides this, BS11 as well as *Butyrivibrio* correlated negatively with 18:0 (Table 4, Figure 3).

**Table 4:** Ruminal bacterial taxa (97% sequence similarity) correlated with 18:2*n*-6, 18:3*n*-3 or related biohydrogenation intermediates.

**Taxon: order/family/genus^3^**	**R^1^^,^^2^**								
	**18:2*n*-6**	**18:3*n*-3**	***c*9, *t*11, *c*15 CLnA**	***c*9, *t*11 CLA**	***t*11 18:1**	***t*10 18:1**	***t*10:*t*11**	**Sum 18:1**	**18:0**
Aeromonadales/Succinivibrionaceae						0.33^$^		0.54^#^	
Aeromonadales/Succinivibrionaceae/*Succinivibrio*			0.31^$^						
Bacteroidales	0.37^#^					−0.38^#^		−0.39^#^	
Bacteroidales/Bacteroidaceae/*Bacteroides*	0.31^$^		0.35^$^						0.35^$^
Bacteroidales/Bacteroidaceae/BF311	0.44^#^	0.42^#^			−0.53^#^	−0.31^$^		−0.45^#^	
Bacteroidales/BS11							−0.49^#^		−0.51^#^
Bacteroidales/Odoribacteraceae/*Butyricimonas*						0.42^#^		0.38^#^	
Bacteroidales/Porphyromonadaceae/*Parabacteroides*			0.32^$^	0.40^#^			0.30^$^		0.40^#^
Bacteroidales/Paraprevotellaceae			−0.49^#^						
Bacteroidales/Paraprevotellaceae/CF231								−0.33^$^	
Bacteroidales/Paraprevotellaceae/*Prevotella*				0.43^#^					
Bacteroidales/Paraprevotellaceae/YRC22					0.30^$^				
Bacteroidales/Prevotellaceae					−0.49^#^		0.35^$^		
Bacteroidales/Prevotellaceae/*Prevotella*				0.37^#^					
Bacteroidales/RF16			−0.51^#^						
Bacteroidales/Rikenellaceae			0.36^#^						
Bacteroidales/S24-7		−0.50^#^		0.38^#^					
Bifidobacteriales/Bifidobacteriaceae/*Bifidobacterium*				0.47^#^		0.50^#^	0.43^#^	0.41^#^	
Clostridiales			−0.35^#^						
Clostridiales/Christensenellaceae		0.47^#^				−0.31^$^			
Clostridiales/Christensenellaceae/*Christensenella*			0.41^#^						
Clostridiales/Clostridiaceae/*Clostridium*	0.31^$^								
Clostridiales/Eubacteriaceae/*Pseudoramibacter eubacterium*	−0.42^#^	−0.30^$^				0.47^#^	0.47^#^		
Clostridiales/Lachnospiraceae					0.34^$^				
Clostridiales/Lachnospiraceae/*Anaerostipes*	0.44^#^	0.44^#^							
Clostridiales/Lachnospiraceae/*Blautia*					−0.31^$^		0.42^#^		
Clostridiales/Lachnospiraceae/*Butyrivibrio*					0.41^#^				−0.57^#^
Clostridiales/Lachnospiraceae/*Coprococcus*		−0.43^#^							
Clostridiales/Lachnospiraceae/*Lachnobacterium*			0.34^$^	0.35^#^					
Clostridiales/Lachnospiraceae/*Lachnospira*	−0.32^$^	−0.44^#^	0.33^$^	0.43^#^	0.35^#^	0.47^#^		0.49^#^	
Clostridiales/Lachnospiraceae/*Pseudobutyrivibrio*									
Clostridiales/Mogibacteriaceae		−0.37^#^						0.36^#^	
Clostridiales/NA/NA		−0.36^#^	0.44^#^						
Clostridiales/Ruminococcaceae		0.32^$^	−0.34^$^	−0.58^#^					0.32^$^
Clostridiales/Ruminococcaceae/NA			*NS*			0.31^$^			
Clostridiales/Ruminococcaceae/*Ruminococcus*			−0.34^$^						
Clostridiales/Veillonellaceae				0.45^#^		0.37^#^			
Clostridiales/Veillonellaceae/NA		−0.33^$^	0.41^#^	0.44^#^		0.42^#^	0.36^#^		
Clostridiales/Veillonellaceae/*Acidaminococcus*			0.53^#^				0.43^#^		0.32^$^
Clostridiales/Veillonellaceae/*Dialister*				0.35^$^		0.63^#^	0.56^#^	0.38^#^	
Clostridiales/Veillonellaceae/*Megasphaera*		−0.31^$^	0.45^#^	0.34^$^		0.48^#^	0.51^#^		0.49^#^
Clostridiales/Veillonellaceae/*Phascolarctobacterium*				0.47^#^	0.34^$^	0.33^$^		0.44^#^	
Clostridiales/Veillonellaceae/*Selenomonas*					0.40^#^				
Clostridiales/Veillonellaceae/*Succiniclasticum*		−0.39^#^			0.32^$^				
Coriobacteriales/Coriobacteriaceae						0.48^#^	0.65^#^		
CW040/F16		0.34^$^		−0.32^$^					
Desulfovibrionales/Desulfovibrionaceae/*Desulfovibrio*							−0.31^$^		
Elusimicrobiales/Elusimicrobiaceae					−0.30^$^				
Enterobacteriales/Enterobacteriaceae						0.38^#^			
Erysipelotrichales/Erysipelotrichaceae/*Allobaculum*	0.38^#^	0.32^$^		0.31^$^	−0.32^$^				
Erysipelotrichales/Erysipelotrichaceae/*Bulleidia*	−0.39^#^					0.32^$^	0.51^#^		
Erysipelotrichales/Erysipelotrichaceae/*Catenibacterium*						0.30^$^			
Erysipelotrichales/Erysipelotrichaceae/*Eubacterium*	−0.45^#^					0.45^#^	0.45^#^		
Erysipelotrichales/Erysipelotrichaceae/L7A_E11		0.30^$^							
Erysipelotrichales/Erysipelotrichaceae/p-75-a5						−0.43^#^	−0.46^#^		
Erysipelotrichales/Erysipelotrichaceae/*Sharpea*						0.31^$^	0.34^$^		
Lactobacillales/Lactobacillaceae/*Lactobacillus*				0.33^$^		0.50^#^	0.57^#^		
Lactobacillales/Streptococcaceae/*Streptococcus*									
Pedosphaerales/R4-41B				−0.43^#^		−0.41^#^	−0.45^#^	−0.32^$^	−0.41^#^
Pirellulales/Pirellulaceae				−0.49^#^		−0.54^#^	−0.49^#^	−0.45^#^	
RF32							0.34^$^		
RF39					−0.43^$^			−0.36^#^	0.34^$^
Rickettsiales							−0.51^#^		
Synergistales/Synergistaceae/NA									
YS2									0.30^$^

*Significances are based on Spearman P-values*.

1 # P < 0.05;

$ *,0.05 ≤ P < 0.10; gray fields, P ≥ 0.10*.

2 *t, trans; c, cis; CLA, conjugated linoleic acid; CLnA, conjugated linolenic acid*.

3 *NA, not assigned*.

## Discussion

Dietary supplementation of products containing DHA previously showed to inhibit the final step of biohydrogenation to 18:0 in the rumen of adult animals, resulting in the accumulation of 18:1 isomers (e.g., Shingfield et al., 2012; Toral et al., 2012; Szczechowiak et al., 2016). However, it is unknown whether similar effects on ruminal biohydrogenation would occur when supplementing DHA-enriched products to ruminants at young age, e.g., during pregnancy or early life. Postnatal supplementation of goat kids with DHA-enriched microalgae altered the rumen FA profile in a similar way as in adult animals, whereas prenatal treatment did not change the rumen FA profile. The ruminal proportion of total 18:1 FA increased from 6.33 to 9.44 g/100 g FA (+3.11 g/100 g FA, +49%) whereas 18:0 decreased from 41.95 to 32.19 g/100 g FA (−9.76 g/100 g FA, −23%) upon postnatal administration of DHA-enriched microalgae. In absolute proportions, the increase in total 18:1 was mostly caused by increased ruminal proportion of *t*11 18:1 (from 0.73 to 1.50 g/100 g FA, +0.77 g/100 g FA, +107%) concurring with other studies (e.g., Shingfield et al., 2012; Toral et al., 2012; Szczechowiak et al., 2016). As opposed to previous reports (e.g., Boeckaert et al., 2007, 2008; Zhu et al., 2016), the absolute increase in *t*10 18:1 of +0.72 g/100 g FA (from 0.63 to 1.35 g/100 g FA, +114%) upon postnatal DHA Gold supplementation was not significant. This could be due to large inter-animal variation ($\bar{x}$ ± SD = 1.00 ± 1.66 g/100 g FA; SEM = 0.597 g/100 g FA) which was also observed in other studies (Kim et al., 2008; Toral et al., 2012). In addition, the amount of DHA supplemented (0.28 g DHA Gold per kg BW corresponding to 0.04 g DHA per kg BW) might have been too limited to induce a general increase in *t*10 18:1. Indeed, supplementation of algae in the rumen of goats induced an increase in ruminal *t*10 18:1 at a dose of 0.19 g DHA per kg BW whereas a dose of 0.06 g DHA per kg BW did not (Zhu et al., 2016). In contrast, supplementation of 0.05 g DHA per kg BW induced an increase in *t*10 18:1 in cows (Shingfield et al., 2012). This discrepancy between goat and cow trials could indicate that both young and adult goats are less prone to diet-induced shifts toward the formation of *t*10 intermediates, in comparison with cows (Toral et al., 2015). The increased ruminal proportion of *c*13 18:1 might indicate that supplementation of DHA-enriched products not only limited *trans* 18:1 but also *cis* 18:1 saturation (e.g., Shingfield et al., 2012; Toral et al., 2012, 2017).

The ruminal proportions of 18:2*n*-6 and 18:3*n*-3 were lower in the postnatally supplemented group, which might indicate that DHA does not cause a general slowdown of the ruminal metabolism of these PUFA. Similar results were observed *in vitro* (Toral et al., 2017) and *in vivo* with cows (Shingfield et al., 2012) and sheep (Toral et al., 2012). However, other *in vivo* studies observed either no differences in 18:2*n*-6, and 18:3*n*-3 (Boeckaert et al., 2007) or higher ruminal proportions of 18:2*n*-6 (Szczechowiak et al., 2016) when supplementing DHA-containing products to cows. Nevertheless, lower relative proportions of 18:2*n*-6 and 18:3*n*-3 can also partly reflect the increase in the total lipid content in the rumen due to supplementation of FA through DHA Gold (dilution effect). If the amounts of 18:2*n*-6 and 18:3*n*-3 are expressed as μg/mL rumen fluid (Supplementary Table 1), no significant difference was observed after postnatal DHA Gold supplementation.

As reported by other authors (Boeckaert et al., 2007; Shingfield et al., 2012; Toral et al., 2017), rumen proportions of *c*9, *t*11 CLA, *t*10, *c*12 CLA, or *c*9, *t*11, *c*15 CLnA were not significantly different between treatments. Others did observe an effect of DHA supplementation on rumen concentrations of CLA and CLnA (Kim et al., 2008; Toral et al., 2012; Szczechowiak et al., 2016). Discrepancies between studies might be related to the amount of DHA supplemented as CLA generally increased with increasing DHA supplementation level. In addition, the time of sampling might also explain the lack of a treatment effect in the current study. Indeed, CLA and CLnA isomers particularly accumulate within a short period after feeding, since thereafter the disappearance rate of these FA exceeds the rate of formation. In the current study, samples of rumen fluid were collected before the morning feeding after an overnight period without access to concentrate and hay.

Hence, postnatal supplementation of DHA-enriched microalgae to goat kids from birth until 12 weeks old affected rumen biohydrogenation in a similar way as in adult animals by inhibition of the final step of biohydrogenation to 18:0. This resulted particularly in increased proportions of *t*11 18:1 rather than a shift to *t*10 intermediates, which suggests that young goats might also be less prone to dietary induced shifts toward the formation of *t*10 intermediates.

Previous studies showed that delivery of FA through the maternal diet during pregnancy can affect ruminal protozoa population, ruminal bacterial community, methane production and reticulorumen weight of the offspring (De Barbieri et al., 2015a,b). Indeed, in the current study, supplementing the maternal diet with DHA-enriched microalgae during pregnancy affected the rumen microbiome of the offspring. In line with the effect of postnatal supplementation on rumen metabolism as discussed in the previous paragraphs, postnatal supplementation of DHA-enriched microalgae also affected the rumen microbiome. Nevertheless, with some taxa (e.g. *Succiniclasticum*, L7A_E11), postnatal treatment with DHA Gold only shifted the relative abundance when goats were not treated prenatally, which could indicate adaptation to a repeated treatment later in life.

The accumulation of different 18:1 isomers and the reduced ruminal proportion of 18:0 after postnatal DHA Gold supplementation may be associated with an inhibitory effect of DHA-enriched microalgae on the proliferation of rumen micro-organisms involved in the reduction of 18:1 FA. *Butyrivibrio proteoclasticus* is the only bacterial species identified to reduce 18:1 FA to 18:0 (Kemp et al., 1975; Wallace et al., 2006; McKain et al., 2010). Indeed, *in vitro* relative abundance of *B. proteoclasticus* decreased when reduction to 18:0 was inhibited by supplementation of a blend of fish oil and soybean oil (Szczechowiak et al., 2016). However, no decrease was observed in the *in vivo* part of that study in accordance with our results and other reports (Huws et al., 2010; Toral et al., 2012; Zhu et al., 2016). Noncultivated *Butyrivibrio, Pseudobutyrivibrio* and other unknown Lachnospiraceae strains might play a role in the final biohydrogenation step (Boeckaert et al., 2008). In the current study, the relative abundance of *Blautia* (family Lachnospiraceae) decreased upon postnatal supplementation of DHA Gold. Furthermore, there was a trend toward negative correlation with *t*11 18:1, potentially indicating that this genus is involved in ruminal 18:0 formation (Huws et al., 2011). In addition, the order RF39 also decreased after postnatal DHA Gold supplementation and was negatively correlated with 18:1 isomers and positively with 18:0. Therefore, genera within this order could also be involved in ruminal 18:0 formation. An alternative explanation is that supplementation of DHA-enriched microalgae reduced the metabolic and perhaps specifically the biohydrogenating activity of *B. proteoclasticus* instead of its proliferation. Metabolic activity may not be proportional to 16S rRNA gene concentration and thus, RNA should be targeted to investigate this hypothesis. However, the current experimental design, sampling at one single time point after overnight fasting, is not appropriate for this purpose. According to Jeyanathan et al. (2016), an alternative explanation is that *B. proteoclasticus* starts to hydrogenate DHA before converting 18:1 isomers to 18:0 because of the higher toxicity of DHA in comparison with 18:1 isomers.

Besides 18:0 as an end product, the biohydrogenation pathways of 18:2*n*-6 and 18:3*n*-3 involve plenty of 18:2 and 18:1 FA intermediates. 18:2*n*-6 is mainly isomerized to *c*9, *t*11 CLA which is further hydrogenated to *t*11 18:1 and ultimately to 18:0 (Bauman and Griinari, 2003; Harvatine et al., 2009). The main biohydrogenation pathway of 18:3*n*-3 involves *c*9, *t*11, *c*15 CLnA, *t*11, *c*15 18:2, and *t*11 18:1 as intermediates (Shingfield and Wallace, 2014). The first ruminal bacterial species reported to produce *c*9, *t*11 CLA was *B. fibrisolvens* (Kepler et al., 1966). According to Wallace et al. (2007), several bacterial species can convert 18:2*n*-6 to *c*9, *t*11 CLA. However, all members of the (*Pseudo*)*butyrivibrio* group form *c*9, *t*11 CLA from 18:2*n*-6 much more rapidly than do other species (Shingfield et al., 2012). *B. fibrisolvens* also further hydrogenates *c*9, *t*11 CLA to *t*11 18:1 (McKain et al., 2010). Previous *in vitro* experiments in our lab (L. Dewanckele, unpublished data) revealed that this bacterial species is also involved in the formation of *c*9, *t*11, *c*15 CLnA, *t*11, *c*15 18:2 and *t*11 18:1 when incubated with 18:3*n*-3. In the current study, *Butyrivibrio* only showed a weak positive correlation with *t*11 intermediates whereas *Pseudobutyrivibrio* showed no correlation with *t*11 intermediates. Together with the study of Zened et al. (2016) in which no correlation was found between *Butyrivibrio* and *t*11 FA, this questions the role of *(Pseudo)butyrivibrio* species in ruminal *t*11 formation. Other as yet uncultured bacteria belonging to genera *Anaerovorax, Prevotella, Lachnospiraceae* Incertae Sedis, *Ruminococcus, Butyrivibrio*, and *Pseudobutyrivibrio, Tanerella* and unclassified Bacteroidales, *Clostridia* and Clostridiales, Ruminococcaceae, Lachnospiraceae, Prevotellaceae and Porphyromonadaceae might be involved in ruminal *t*11 formation (Huws et al., 2011). This study confirmed this potential role for e.g., *Parabacteroides* (Porphyromonadaceae family), *Prevotella*, the families Rikenellaceae and S24-7 (Bacteroidales), *Christensenella* and *Lachnospira* (Clostridiales).

Dietary supplementation of DHA-enriched supplements could induce a shift from the main biohydrogenation pathway toward the formation of *t*10 intermediates (e.g., *t*10, *c*12 CLA, *t*10, *c*15 18:2, and *t*10 18:1). However, the ruminal bacteria involved in *t*10 formation remain unclear. In this study, *Megasphaera* correlated positively with *t*10 18:1 and with the ratio of *t*10 to *t*11 intermediates. *M. elsdenii* was found to convert *in vitro* 18:2*n*-6 to *t*10, *c*12 CLA (Kim et al., 2002). Nevertheless, Maia et al. (2007) observed no production of *t*10, *c*12 CLA by this bacterial species. *In vitro* studies by the group of Wallace (Wallace et al., 2007) further indicated that *Propionibacterium acnes* is a producer of *t*10, *c*12 CLA, which is the end product of its 18:2*n*-6 metabolism (McKain et al., 2010). This was confirmed in our previous *in vitro* experiments (Dewanckele et al., 2017). However, ruminal abundance of this species is very low (Shingfield et al., 2012) and in the current study, it was even not observed. *Lactobacillus* spp. have also been shown to produce *t*10, *c*12 CLA *in vitro* (Alonso et al., 2003; Renes et al., 2017), which was highlighted in the current study. However, to what extent this microorganism plays a role in ruminal biohydrogenation remains unclear. Moreover, we validated that *Dialister* was positively correlated with *t*10 FA (Zened et al., 2016). *In vitro* experiments with pure cultures are required to confirm the capacity of this genus to produce *t*10 isomers. Other genera positively correlated with ruminal *t*10 isomers according to this study are: *Bifidobacterium, Sharpea, Pseudoramibacter eubacterium, Eubacterium*, and undefined genera belonging to the family Coriobacteriaceae. However, none of them have been identified as major *t*10 FA producers (e.g., Devillard et al., 2007; McIntosh et al., 2009; Gorissen et al., 2010). Nevertheless, production of *trans* isomers is species- and strain-dependent (Gorissen et al., 2010).

Correlations between different biohydrogenation intermediates and rumen bacteria are comparable between our results, which were based on young goats, and other reports, which were based on adult animals. Hence, these extensive comparisons made between current results and literature data indicate that associations between biohydrogenation intermediates and rumen bacteria in goat kids of 12 weeks old align with former observations in adult ruminants.

## Conclusion

Postnatal supplementation of goat kids from birth until 12 weeks old with DHA-enriched microalgae affected rumen biohydrogenation in a similar way as in adult animals by inhibition of the final step of biohydrogenation to 18:0. This resulted particularly in increased ruminal proportions of *t*11 18:1 rather than a shift to *t*10 intermediates, which suggests that young goats, just as adult ones, might be less prone to dietary induced shifts toward the formation of *t*10 intermediates, in comparison with cows. Higher abundance of *Butyrivibrio* when the reduction to 18:0 was inhibited is surprising as they have been identified as the most important biohydrogenating bacteria. *Blautia* abundance was positively correlated with 18:0 accumulation, whereas *Lactobacillus* spp. *Dialister* spp. and *Bifidobacterium* spp. were more abundant in situations with greater *t*10 accumulation. Extensive comparisons made between current results and literature data indicate that current associations between biohydrogenation intermediates and rumen bacteria in young goats align with former observations in adult ruminants.

## Author contributions

VF, AR-G, and SD conceived and designed the animal experiment. LD performed the chemical analysis of LCFA. LD, JJ, and EH-S performed the bacterial community analysis. LD analyzed the data. LD, BV, and VF discussed and interpreted the obtained data. LD wrote the manuscript. BV and VF contributed to the writing of the manuscript. All authors read and approved the final manuscript.

### Conflict of interest statement

The authors declare that the research was conducted in the absence of any commercial or financial relationships that could be construed as a potential conflict of interest.

## Abbreviations

BW: body weight
*c*: *cis*
CLA: conjugated linoleic acid
CLnA: conjugated linolenic acid
DHA: docosahexaenoic acid
EPA: eicosapentaenoic acid
FA: fatty acid
FAME: fatty acid methyl ester
LCFA: long-chain fatty acid
OTU: operational taxonomic unit
PUFA: poly-unsaturated fatty acid
SD: standard deviation
*t*: *trans*.
