# Supplementary material for: Rumen Biohydrogenation and Microbial Community Changes Upon Early Life Supplementation of 22:6n-3 Enriched Microalgae to Goats
Source: Front Microbiol. 2018 Mar 27;9:573. doi: 10.3389/fmicb.2018.00573 (PMC5880937; doi:10.3389/fmicb.2018.00573)
Supplement: Supplementary file 1 [file DataSheet1.docx]

Supplementary Material

Rumen Biohydrogenation and Microbial Community Changes upon Early Life Supplementation of 22:6*n*-3 Enriched Microalgae to Goats

Lore Dewanckele, Bruno Vlaeminck, Emma Hernandez-Sanabria, Alexis Ruiz-González, Sieglinde Debruyne, Jeyamalar Jeyanathan, Veerle Fievez*

*** Correspondence:** Veerle Fievez: Veerle.Fievez@UGent.be

# Supplementary Figures and Tables

## Supplementary Figures

**
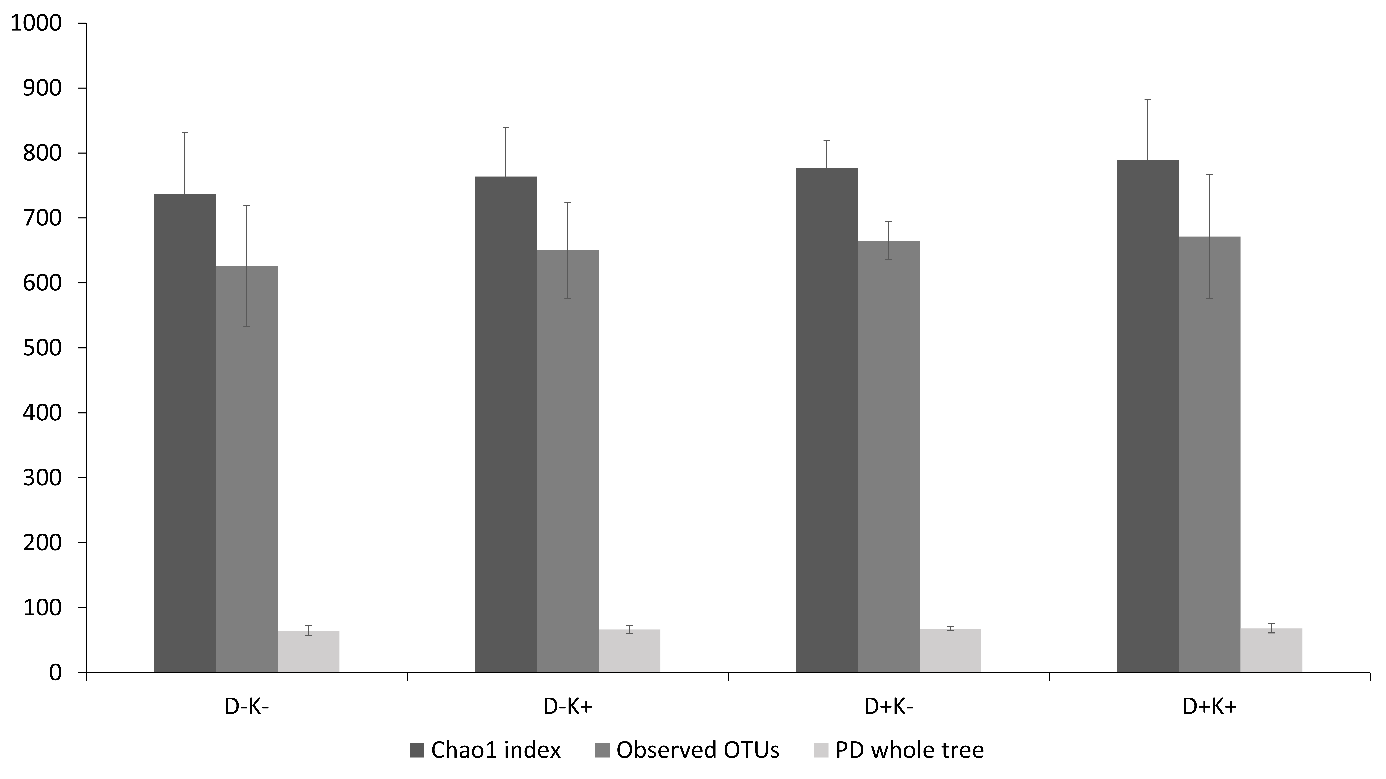
**

**Supplementary Figure 1.** Alpha diversity in the rumen fluid in different experimental groups. Data are presented as mean ± SD. No significant differences (*P* ≥ 0.05) were observed. D, doe; K, kid; ^+^, supplemented with DHA Gold (0.28 g per kg BW); ^-^, no DHA Gold supplementation.

## Supplementary Tables

**Supplementary Table 1.** Effect of prenatal and/or postnatal treatment of goat kids with DHA Gold on the concentration of long-chain fatty acids (µg/mL) in rumen fluid

| Fatty acid ^(3)^ | Experimental group ^(1)^ | | | | SEM ^(2)^ | *P*-value | | |
| --- | --- | --- | --- | --- | --- | --- | --- | --- |
|  | D^-^K^-^  (n = 8) | D^-^K^+^  (n = 8) | D^+^K^-^  (n = 8) | D^+^K^+^  (n = 8) |  | Prenatal treatment (D) | Postnatal treatment (K) | D × K |
| 14:0 | 21.77 | 60.95 | 33.72 | 54.34 | 5.882 | 0.623 | < 0.001 | 0.079 |
| 16:0 | 221.83 | 339.33 | 209.42 | 290.92 | 26.858 | 0.364 | < 0.001 | 0.380 |
| 18:0 | 649.98 | 645.12 | 550.88 | 549.51 | 97.504 | 0.424 | 0.966 | 0.981 |
| *t*6 18:1 + *t*7 18:1 + *t*8 18:1^*^ | 3.14 | 9.71 | 2.17 | 7.76 | 2.377 | 0.149 | 0.002 | 0.411 |
| *t*9 18:1^*^ | 1.04 | 5.83 | 0.87 | 4.46 | 1.058 | 0.266 | < 0.001 | 0.496 |
| *t*10 18:1^*^ | 11.45 | 20.40 | 7.74 | 39.75 | 16.352 | 0.372 | 0.125 | 0.671 |
| *t*11 18:1^*^ | 9.01 | 30.49 | 10.99 | 27.68 | 8.136 | 0.804 | 0.001 | 0.264 |
| *t*12 18:1 | 2.93 | 10.28 | 1.56 | 6.25 | 1.673 | 0.129 | 0.003 | 0.440 |
| *c*9 18:1 + *t*13 18:1 + *t*14 18:1 | 37.60 | 55.06 | 30.74 | 42.42 | 7.410 | 0.209 | 0.069 | 0.702 |
| *c*11 18:1 + *t*15 18:1 | 17.95 | 36.79 | 21.28 | 27.25 | 4.178 | 0.471 | 0.010 | 0.146 |
| *c*12 18:1 | 5.07 | 5.55 | 5.10 | 4.60 | 0.770 | 0.562 | 0.994 | 0.537 |
| *c*13 18:1 | 1.33 | 7.02 | 3.57 | 5.99 | 3.402 | 0.822 | 0.013 | 0.272 |
| *c*14 18:1 + *t*16 18:1 | 5.33 | 8.47 | 1.72 | 4.68 | 3.052 | 0.165 | 0.035 | 0.947 |
| Sum 18:1 | 989.74 | 1236.32 | 878.44 | 1064.31 | 141.22 | 0.404 | 0.077 | 0.792 |
| *c*9, *t*11 CLA | 1.69 | 3.82 | 1.79 | 3.49 | 1.249 | 0.932 | 0.115 | 0.855 |
| *t*10, *c*12 CLA | 0.35 | 0.32 | < 0.01 | < 0.01 | 0.236 | 0.180 | 0.943 | 0.943 |
| 18:2*n*-6 | 42.34 | 40.20 | 34.17 | 28.13 | 9.243 | 0.397 | 0.510 | 0.752 |
| *c*9, *t*11 CLA + *t*11 18:1^*^ | 10.70 | 34.32 | 12.77 | 31.18 | 8.679 | 0.766 | 0.002 | 0.271 |
| *t*10, *c*12 CLA + *t*10 18:1^*^ | 11.80 | 20.72 | 7.74 | 39.75 | 16.353 | 0.343 | 0.123 | 0.672 |
| 18:3*n*-3 | 8.64 | 7.88 | 8.84 | 8.70 | 1.416 | 0.706 | 0.720 | 0.805 |
| *c*9, *t*11, *c*15 CLnA | 1.50 | 2.98 | 1.39 | 1.13 | 0.694 | 0.275 | 0.212 | 0.083 |
| 22:5*n*-6 | < 0.01 | 26.31 | 1.40 | 29.11 | 4.008 | 0.382 | < 0.001 | 0.910 |
| 22:6*n*-3 | < 0.01 | 69.82 | 4.23 | 76.54 | 11.110 | 0.343 | < 0.001 | 0.980 |
| Total fatty acids | 1435.11 | 1918.40 | 1336.13 | 1705.66 | 170.74 | 0.451 | 0.007 | 0.678 |

^(1)^ D, doe; K, kid; ^+^, supplemented with DHA Gold (0.28 g per kg BW); ^-^, no DHA Gold supplementation.

^(2)^ SEM, standard error of the mean.

^(3)^ *t*, *trans*; *c*, *cis*; CLA, conjugated linoleic acid; CLnA, conjugated linolenic acid.

^*^ Reported *P*-values are the *P*-values from the logarithm.
